# Nurses’ contributions to reproductive rights in family planning: a qualitative study

**DOI:** 10.1590/0034-7167-2024-0542

**Published:** 2025-12-15

**Authors:** Joselaine Rigue, Leandro da Silva de Medeiros, Claudia Zamberlan, Márcia Noélia Pestana Santos, Dirce Stein Backes, Naiana Oliveira dos Santos, Cristina Saling Kruel

**Affiliations:** I Universidade Franciscana. Santa Maria, Rio Grande do Sul, Brazil; II Escola Superior de Enfermagem de Coimbra. Coimbra, Portugal; III Universidade Federal de Santa Maria. Santa Maria, Rio Grande do Sul, Brazil

**Keywords:** Sustainable Development, Nursing, Family Planning Policy, Reproductive Health, Maternal-Child Health Services., Desarrollo Sostenible, Enfermería, Planificación Familiar, Salud Reproductiva, Salud Materno-Infantil.

## Abstract

**Objective:**

To understand how primary care nurses understand and integrate reproductive rights in the context of family planning.

**Method:**

Action research developed between September and November 2023 with five nurses from a Primary Care Health Unit in the central region of Rio Grande do Sul. Data were collected through focus groups and analyzed using the content analysis technique.

**Results:**

Nurses highlighted reproductive autonomy, with an increase in the demand for tubal ligations; personalized education on contraception; the socioeconomic impact on access to family planning; and challenges related to male inclusion, with strategies aimed at co-responsibility in planning.

**Final considerations:**

Nurses play an essential role in promoting reproductive rights. By respecting individualities and the social context, nurses promote shared responsibility and strengthen female autonomy, which are aligned with the Sustainable Development Goals that aim at gender equality and reducing inequalities.

## RESUMEN

**Objetivo:**

Comprender cómo las enfermeras de atención primaria plantean los derechos reproductivos en el contexto de la planificación familiar.

**Método:**

Investigación-acción realizada entre septiembre y noviembre de 2023 con cinco enfermeras de una Unidad Básica de Salud de Río Grande del Sur. Los datos se recogieron a través de grupos focales y se analizaron mediante el análisis de contenido.

**Resultados:**

Las enfermeras destacaron la autonomía reproductiva, con aumento de la demanda de ligaduras de trompas; la educación anticonceptiva personalizada; el impacto socioeconómico del acceso a la planificación familiar; y los retos sobre la inclusión masculina, con estrategias dirigidas a la corresponsabilidad en la planificación.

**Consideraciones finales:**

Las enfermeras desempeñan un papel esencial en la promoción de los derechos reproductivos, respetando la individualidad. Promueven la responsabilidad compartida y refuerzan la autonomía femenina, alineadas a los objetivos del Desarrollo Sostenible, con miras a la igualdad de género y a la reducción de desigualdades.

## INTRODUCTION

Health and social well-being are significantly influenced by sexual and reproductive rights, which are reflected in the Sustainable Development Goals (SDGs) established by the United Nations (UN), which aim at quality health (SDG 3), quality education (SDG 4), gender equality (SDG 5) and reduction of inequalities (SDG 10)^(1)^. The analysis of these rights from different perspectives, including legislation, public policies and social movements, is crucial to understanding the advances and challenges in achieving universal access to reproductive health and sexual freedom without coercion or discrimination^(2)^.

The SDGs constitute a global agenda established by the UN in 2015, with the purpose of eradicating poverty, protecting the environment and promoting well-being and opportunities for all in a fair and sustainable manner^(1,3)^. It comprises 17 goals and 169 targets, the SDGs cover critical challenges such as poverty, health, education, gender equality, and environmental sustainability^(1)^. In this way, they serve as a blueprint for joint actions between governments, civil society, the private sector and individuals, aiming to build a more equitable and prosperous world^(1,3)^. Health professionals, especially nurses, have an essential role in achieving the SDGs due to their commitment to care and global health^(4)^.

Health care, with a special emphasis on primary care, plays a decisive role in the realization of reproductive rights, especially when health services are inclusive, equitable and user-centered. The Family Planning Law of 1996, for example, was a milestone in the consolidation of family planning as a right and in guaranteeing access to contraceptive methods and information on reproductive health in Brazil^(5)^. These initiatives have contributed to the improvement of health and well-being indicators and to progress towards the SDGs, such as the reduction of unplanned pregnancy rates and maternal and neonatal mortality, in addition to the prevention of Sexually Transmitted Infections (STIs) and financial impacts on families^(6,7)^.

However, despite advances, the full implementation of laws and policies is still not a reality in all regions of the country, resulting in significant disparities in access to and guarantee of sexual and reproductive rights^(8)^. Sociodemographic disparities directly influence family planning assistance and result in unequal access to health services, leading to higher rates of unplanned pregnancies and STIs^(9)^. In addition, controversial issues, such as the legalization of abortion and the reproductive rights of LGBTQIA+ people, face political and social resistance, limiting access to essential services^(10)^.

Public policies aimed at sexual and reproductive health must be developed with decentralized strategies, operating within the scope of Primary Health Care (PHC) to promote comprehensive and equitable care^(11)^. Strengthening family planning is essential to transform these rights into a reality accessible to all, reflecting a commitment to comprehensive health and human dignity and the principles of the SDGs.

In this approach, nursing occupies a strategic position in the promotion of reproductive rights, particularly in the context of family planning. Nurses are often the first health professionals to interact with users, play a key role in education and counseling, and provide essential reproductive health services^(12)^. This privileged position enables them to act as advocates for users’ rights, ensuring access to quality information and services, respecting individual autonomy and choices, and advancing the achievement of the SDGs^(13)^.

Nursing professionals also play an active role in providing direct reproductive health care, which includes administering contraceptives, performing preventive exams, monitoring during pregnancy and postpartum, and treating STIs^(14)^. Therefore, nursing’s role in family planning and reproductive rights contributes to health promotion, education, and care, and is essential for the development of public policies and evidence-based health practices, thus shaping the health field in the country.

## OBJECTIVE

Understanding how primary care nurses understand and integrate reproductive rights in the context of family planning.

## METHOD

### Ethical aspects

This study followed the recommendations of the National Health Council (NHC) Resolution No. 466/2012 and was approved by the Research Ethics Committee of the proposing institution. Free and Informed Consent was obtained from all participants involved in the research in writing. To ensure the anonymity of the participants, their statements were identified by the letter “N” for Nurse, followed by a numerical number, from N1 to N5.

### Theoretical-methodological framework

Anchored in the UN SDGs, which address challenges such as health, education and gender equality, this research focuses on family planning in primary health care. Family planning in primary care is crucial to advancing goals related to quality health (SDG 3), quality education (SDG 4), gender equality (SDG 5) and reduction of inequalities (SDG 10), contributing directly to population sustainability and community well-being, thus aligning with global efforts to achieve the 2030 Agenda Goals. Nursing professionals are recognized for their strategic contribution in promoting these goals, especially in the context of primary health care^(4,15)^.

### Study design

An action research was carried out in a Primary Care Health Unit in a city in the central region of the state of Rio Grande do Sul. Action research has a participatory nature, democratic impulse and contributes to social change, it develops from a need identified by the collective, provoked by the researcher, involving efforts, analyses and reflections of the participants in the observed context^(16,17)^. The study report followed the guidelines of the Consolidated Criteria for Reporting Qualitative Research (COREQ) to ensure methodological rigor and transparency^(18)^.

### Study Setting

The study was conducted at a Primary Care Health Unit (PCHU) in the center of the state of Rio Grande do Sul, chosen because it was the main researcher’s workplace and because it offered a favorable scenario for the investigation, due to the high prevalence of unplanned pregnancies and short intrapartum intervals. To minimize the risk of bias, the main researcher followed the COREQ guidelines (Domain 1 - Relationship with participants), maintaining a reflective stance and ensuring that the dynamics of the focus groups were facilitated by an independent moderator. The researcher obtained authorization from the management to implement the nursing consultation on family planning.

The PCHU in question has two Primary Care Teams (PCT) and two Family Health Strategy (ESF) teams. The nursing team at the PCTs is composed of two nurses and four nursing technicians for both teams; and the ESF has two nurses and three nursing technicians. The PCHU also has a first-year resident nurse in obstetric nursing and a nursing professor from two universities in Rio Grande do Sul. Therefore, the team is made up of six nurses, one of whom is the researcher.

### Data Sources

All five nurses from the PCHU, including a resident, a faculty member, and nurses from the Primary Care and Family Health Strategy teams, participated in the study. Inclusion criteria were working on PCHU teams and having time to participate in the three focal meetings. Exclusion criteria were being on supervision, on vacation, on medical leave, and/or unavailable to participate in the three meetings. The participants were approached personally by the lead researcher, who presented the research objectives and methods, explaining the importance of each of their participation for the study. Initial meetings were held to clarify doubts and obtain the nurses’ free and informed consent. This direct approach facilitated the creation of an environment of trust and collaboration between the researcher and the participants.

### Data collection and organization

Data were collected using the focus group (FG) technique, which allows for in-depth discussions related to a specific topic of common interest to participants^(19)^. Three focus groups were held between September and November 2023, with each meeting lasting an average of one and a half hours. The days and times of the meetings were scheduled in advance with the participants and were held after the afternoon shift.

The meetings were held in a meeting room at the PCHU, with chairs arranged in a circle to encourage discussion and interaction among participants. The focus group meetings were organized with the participation of the main researcher, who served as coordinator; a moderator, who was responsible for the discussions with the coordinator; and two scientific initiation scholarship holders, who had been previously trained and were responsible for recording audio on a smartphone (iPhone 8 Plus) and taking notes.

The focus group was conducted based on a discussion trigger guide, which included questions such as: How do you understand and integrate reproductive rights in the context of family planning? What are the main challenges you face in promoting these rights? How do users’ socioeconomic conditions influence access to family planning? What strategies do you use to personalize guidance on contraceptive methods? How have recent legislative changes impacted your work in relation to reproductive rights? The clarity and relevance of the issues were discussed with the Research Group, composed of the main researcher of the research (nurse), an auxiliary researcher (nurse), a PhD researcher (nurse), a scientific initiation scholarship holder (psychology student) and the research supervisor (psychologist).

During the meetings, the information mentioned was verified and recorded, with a final reading carried out at the end of each meeting, allowing the participants the opportunity to complement and clarify some information and, above all, to receive the results of the research.

### Data analysis

The recorded data were transcribed in full and submitted to Content Analysis^(20)^, following the steps of pre-analysis, exploration of the material and treatment of the results. In the pre-analysis, the collected data were read fluently, seeking a first impression and representation of the messages; in the exploratory phase, the material was coded into categories through excerpts from the records; and in the third and final stage, treatment of the results, the data were treated in a way that could be meaningful, through critical and reflective evaluation.

## RESULTS

The study resulted in the participation of five nurses, aged between 25 and 48 years old. Their professional experience at the PCHU ranged from 11 months to 15 years. All of them have a lato sensu postgraduate degree, specialization type, and two have stricto sensu postgraduate degrees, master’s and doctorate, respectively. Two nurses also work in another service in hospital care. From the organized and analyzed data, three categories emerged: Health education on sexual and reproductive health; Social vulnerability and reproductive rights; and Nurses’ perceptions and contributions to reproductive rights.

### Health education on sexual and reproductive health

In this category, data emerged on how nurses promote health education, with a focus on personalizing guidance, diversifying contraceptive methods, and including family planning in different care contexts.

*During the nursing consultation, we talk about what is available, we see the patient’s preferences and the possibilities. There is the issue of the high incidence of those who miss the deadline to take that injection, so we need to do the rapid pregnancy test, or a BHCG. So, considering spontaneous demand, this would be the moment when we would have a conversation, provide guidance, on this topic.* (N2)

*In the prenatal period, in the last trimester, when we start talking about family planning, we talk about their life plans, whether having another pregnancy is a plan, soon or not, and about possible contraceptive methods to be used, whether they are exclusively breastfeeding or not, in the case of a pregnant woman with HIV. These are the issues that we focus on when we think about family planning.* (N1)

*I think my speech is more focused on this part* [of contraception]*, providing guidance, asking if there is anything they prefer. During a nursing consultation, when you are doing a rapid test, you are asking the woman how her contraceptive is going, or if she is using any other method. Sometimes she comes with a complaint more related to menopause and climacteric. The nursing consultation is very broad.* (N2)

Challenges in communication and understanding about contraceptive methods were especially noted in contexts of psychosocial vulnerability.

*Psychological and social issues, because there are some who will not adhere to that method, even if you explain, explain, explain. Sometimes you have to pay more attention, because they* [users] *do not have the capacity to understand as much as we think we are passing on to them. So this is a big issue, we think they understand and then they come to the next appointment and they do not understand anything about how to use the pill, or what it will do to their lives.* (N5)

### Social vulnerability and reproductive rights

Nurses highlighted how social vulnerability affects access to and effectiveness of family planning, recognizing the interrelationship between socioeconomic conditions and reproductive rights.

*I think that when we talk about family planning,* [...] *we think about the importance, as professionals who work in women’s health, of talking about these sexual and reproductive rights, and that there is an important issue* [...] *which is vulnerability, the situation of vulnerability to which women are exposed* [...] *this vulnerability that is individual, that is social, that is drug users, that suffers violence, in short, that these issues also affect family planning.* (N1)

*I think that, due to vulnerability, people think it is normal to lose several children* [...] *and they do not seek help beforehand, and this also reflects a little on family planning as a whole, because they also do not seek help when they need to prevent pregnancy.* [...]*they do not seek help because they have this social and family vulnerability as well.* (N5)

Discussions on socioeconomic dimensions highlighted the ongoing challenge in promoting reproductive rights.

*We know about the vulnerability issues related to poverty, right? Few financial resources.* (N3)

*Our women and children are very vulnerable.* [...] *An email arrived to follow up on a case of a homeless woman.* (N4)

*We had a case, she had 4 abortions and she wanted, planned and really wanted a pregnancy. We know about the vulnerability issues related to poverty* [...]*. But there are reproductive rights, she really wanted to be a mother and she also had that right. Even though, sometimes, we don’t agree with them, because we know that, in a little while, she will be exposed to greater vulnerability, they have to have the right.* (N5)

### Nurses’ perceptions and contributions to reproductive rights

Nurses’ contributions to ensuring reproductive rights were highlighted, addressing both legislative changes and women’s autonomy in their reproductive decisions.

*We know that many women* [...] *are still subjected to a man’s decisions, so they are gaining this autonomy that is now supported by the change in the law.* [...] *There is more demand, because now if I am 21, if I have two children, I have autonomy. Before, I depended on my partner’s approval, anyway, it was truncated.* (N2)

*We have several situations where each partner has one child, who is the partner at the time, so they were held hostage because they did not meet the requirements* [for tubal ligation]*, and in my opinion that is not fair, because it is her body, she* [the woman] *is the one who has to decide.* (N4)

*She is pregnant as a result of rape, let’s see if she will have access to this planning, this tubal ligation that she wants so much, because the law, as always, is very nice* [...]*. If it were in the past, she would have to look for this man, to give the ‘ok’, imagine having to look for her rapist to get permission.* (N4)

*I also talk about methods, when it comes to pregnancy, we talk about an IUD* [Intrauterine Device]*, a tubal ligation, if she wants to. We talk a little with the husband* [...] *then we evaluate this, talk about the methods.* (N5)

*There is a lot of demand for tubal ligation and we are already referring men for tubal ligation and vasectomy. So as not to overload the woman, because it is always the woman, right? So, we already make referrals to women’s health, we already provide guidance to both, to share responsibility, because family planning is not something only for women.* (N4)

*At the moment we are making more referrals for tubal ligation, I think it is due to the legislation that has become more facilitating.* (N2)

## DISCUSSION

### Health education on sexual and reproductive health

In PHC, nurses play a central role in education and communication about sexual and reproductive health. Nursing consultations reveal a strategic approach that emphasizes individualized care and ongoing support for the use of contraceptive methods. Routine consultations and prenatal sessions provide opportunities to discuss family planning and select appropriate contraceptive methods. The individualized guidance provided by nurses N2 and N1 highlights the need for a personalized approach that involves exploring contraceptive options, understanding users’ preferences, and monitoring adaptation to the chosen methods. The practice reflects a base of knowledge, experience, and mutual respect, which are essential for building a relationship of trust and effective communication in sexual health^(21)^. The emphasis on life projects and considering individual circumstances, such as breastfeeding or specific conditions such as HIV, are crucial to ensuring reproductive rights, reinforcing the literature that emphasizes the role of the nurse as a mediator between the community and health services^(22)^. Regardless of the methodological approach used, educational activities should encourage active participation and the exchange of information, strengthening the bonds between users and health professionals^(23)^. The collaborative approach and the comprehensive approach are fundamental for comprehensive care for women’s health^(24)^, remaining a vital component for achieving the SDGs, as well as the knowledge necessary to make informed decisions about their sexual and reproductive lives, positively impacting their autonomy, quality of life and consequently their health.

Adverse socioeconomic factors, low educational levels and a diminished perception of social status can result in reduced health literacy. Such circumstances require professionals to pay extra attention and strengthen communication strategies to ensure understanding and appropriate use of contraceptive methods^(25,26)^.

The role of nurses in sexual and reproductive health education goes beyond the transmission of information, including understanding individual circumstances. To face the challenges of effective communication, personalized monitoring is necessary, especially in contexts of vulnerability^(27)^.

### Social vulnerability and reproductive rights

Social vulnerability includes people with the same peculiarities of being susceptible to suffering some type of injury that goes beyond physical and moral. It is known that women in social vulnerability have their rights (re)denied on a daily basis. In this sense, effective public and affirmative policies for women are necessary, resulting in a new way of viewing women as citizens, inserted in the family and society, as holders of rights, obligations, prerogatives and duties^(28)^.

Social vulnerability, marked by the denial of rights, impacts women’s ability to make informed decisions about sexual and reproductive health. The normalization of adverse events, such as abortion and infant mortality, and late access to health services reveal the need for educational strategies adapted to women’s socioeconomic conditions^(9)^. Social vulnerability combined with reproductive rights can significantly impact health practices, creating barriers that hinder access to health care. These barriers perpetuate a cycle of inequality that not only affects women’s health but also impedes progress towards the SDGs. Addressing these vulnerabilities requires an intersectoral and integrated approach that involves reducing social and economic inequalities, promoting gender equality, and strengthening partnerships to ensure that health service users are able to make informed choices in accordance with their reality.

The difficulty in accessing health information and services reflects a barrier to the exercise of reproductive rights, especially in vulnerable groups, where family planning actions need to be intensified, which requires educational strengthening work through PHC^(29)^. The study participants question how sociocultural issues impact health and mention that, among the population served, there are situations of violence, sexism, and drug use, and recognize how these situations should be considered relevant. This highlights the need to strengthen social policies in the health area and that the challenges are at various levels where public authorities’ actions must respond to the comprehensive promotion of the population’s sexual and reproductive rights^(30)^. The right to motherhood, even in contexts of vulnerability, is an ethical issue, and it is the State’s duty to guarantee social protection and adequate health care conditions^(31)^.

### Nurses’ perceptions and contributions to reproductive rights

The study highlights the nurses’ perception that family planning is essential to ensure women’s reproductive rights, highlighting female autonomy in decisions regarding reproductive health as a fundamental right. Recent legislative changes, in line with international treaties, have expanded women’s access to these rights, increasing the demand for reproductive procedures and reflecting the progressive emancipation of women^(32)^. Such perceptions corroborate a study that identified an increase in the number of tubal ligations performed between 2019 and 2022, with further growth in 2023, and attests that the approval of the new tubal ligation law empowers women in relation to their reproductive rights^(33)^.

The changes in legislation highlight the historical transition towards gender equality, eliminating obsolete requirements that restricted women’s autonomy, such as spousal authorization for tubal ligation procedures^(34)^. The topic involves ethics, politics, religion, customs, social and demographic issues, which are topics of discussion on the global stage. The changes made to Law 14,443 represent a milestone in social movements seeking greater autonomy for women and the pursuit of gender equality. In particular, the repeal of the requirement for express consent by the spouse represents a major advance in women’s social achievements^(35)^.

However, challenges persist in health education and equitable access to family planning services, with nurses emphasizing the need to involve men in consultations to promote co-responsibility. The literature suggests that the rates of contraceptive methods with the greatest representation of social choice are tubal ligation and that this preference may be related to the lack of health education, coverage issues, and the capture of the male public in reproductive planning services^(36,37)^. Joint actions are essential to ensure the reproductive health and well-being of the couple, with the National Health System (SUS^1^) offering specific contraceptive methods for men, such as vasectomy and male condoms, promoting gender equity in reproductive planning^(38-40)^.

It is imperative to adopt a broader approach that considers sexual and reproductive health throughout the life cycle, actively involving men to overcome gender inequalities and promote co-responsibility^(41)^. These perceptions corroborate another study, in which the nurses interviewed also perceive that women are victims of gender inequalities, of exclusive responsibility for protection against STIs and contraceptive measures, with a consequent lack of partner monitoring and blaming women for unwanted pregnancies^(42)^. Joint parental responsibility has beneficial effects on children’s development and the strengthening of family ties, with health professionals encouraging male participation from prenatal consultations to postpartum care^(39)^.

However, the nurses’ perceptions demonstrate a cisheteronormative matrix, with gaps in meeting the needs of LGBTQIA+ people, who face additional barriers in accessing family planning services. It is necessary to expand approaches to include a diverse understanding of gender identities and sexual orientations, ensuring that reproductive rights are accessible to all. Even with the problems of social, structural, human, financial and management context, the role of nurses still stands out as essential professionals for promoting health and providing comprehensive care to the population^(43)^. Nevertheless, all these findings show that there is an indisputable need for government action to guarantee reproductive rights, by protecting people’s health and autonomy, promoting gender equality and eliminating legal and social barriers that may limit access to these rights.

### Study limitations

The research was conducted in only one health unit, limiting the number of participants and the extension of the results to other contexts. Specific cultural and contextual factors should be considered when transposing the results to different realities.

### Contributions to the area of nursing, maternal and child health and public policies

The research suggests improvements in public policies, with emphasis on the inclusion of men in family planning and co-responsibility in contraception, in line with the SDGs. Nursing professionals are essential to implementing these changes, influencing the formulation of efficient policies. Educational strategies and public policies that promote reproductive rights reinforce the strategic role of nursing in promoting health and well-being, in accordance with the principles of the SDGs.

## FINAL CONSIDERATIONS

This research provided a qualitative analysis of the understanding and integration of reproductive rights by nurses working in primary care, within the scope of family planning. The professionals highlighted the need to individualize women’s health care, emphasizing the importance of addressing such issues in a personalized manner, respecting the preferences and needs of users. The challenges of communication and understanding were highlighted, where improving health literacy emerges as a strategy to ensure the clarity and accessibility of information.

The influence of social vulnerability on reproductive rights was recognized, highlighting that socioeconomic conditions are decisive for promoting equity in access to reproductive health care. The nurses observed that recent legislative reforms have strengthened female reproductive autonomy, reflected in the increased demand for procedures such as tubal ligation. However, obstacles were identified in the operationalization of these policies, underscoring the need to meet women’s expectations. The preference for specific contraceptive methods points to gaps in health education and challenges in male inclusion in reproductive planning services. The importance of integrating men into family planning consultations is highlighted, aiming at an equitable distribution of contraceptive responsibilities.

Finally, this research highlights the relevant contribution of primary care nurses in promoting the SDGs, with an emphasis on the goals that address quality health (SDG 3), quality education (SDG 4), gender equality (SDG 5) and the reduction of inequalities (SDG 10). By promoting women’s autonomy, respecting individualities and encouraging shared responsibility in contraception, nurses play a fundamental role in implementing practices aligned with the global goals for sustainable development. The importance of robust public policies and educational initiatives focused on strengthening capacity in reproductive rights for health professionals and community members is highlighted, thus reinforcing the strategic role of nursing in achieving these global goals.

## Data Availability

Available research data is in the body of the article.

## Footnotes

1 SUS stands for “*Sistema único de saúde*”, a very well-known acronym in the Brazil. Therefore, we chose to keep SUS throughout our paper.
