# Supplementary material for: Nurses’ contributions to reproductive rights in family planning: a qualitative study
Source: Rev Bras Enferm. 2025 Dec 15;78(5):e20240542. doi: 10.1590/0034-7167-2024-0542 (PMC12707846; doi:10.1590/0034-7167-2024-0542)
Supplement: Translation

# Translation: Contribuições de enfermeiras para os direitos reprodutivos no planejamento familiar: estudo qualitativo

**DOI:** 10.1590/0034-7167-2024-0542pt

**Authors:** Joselaine Rigue, Leandro da Silva de Medeiros, Claudia Zamberlan, Márcia Noélia Pestana Santos, Dirce Stein Backes, Naiana Oliveira dos Santos, Cristina Saling Kruel

**Affiliations:** I Universidade Franciscana. Santa Maria, Rio Grande do Sul, Brasil; II Escola Superior de Enfermagem de Coimbra. Coimbra, Portugal; III Universidade Federal de Santa Maria. Santa Maria, Rio Grande do Sul, Brasil

**Keywords:** Desenvolvimento Sustentável, Enfermagem, Planejamento Familiar, Saúde Reprodutiva, Saúde Materno-Infantil.

## Abstract

**Objetivo:**

Compreender como enfermeiras da atenção primária compreendem e integram os direitos reprodutivos no contexto do planejamento familiar.

**Método:**

Pesquisa-ação desenvolvida entre setembro e novembro de 2023 com cinco enfermeiras de uma Unidade Básica de Saúde da região central do Rio Grande do Sul. Os dados foram coletados por meio de grupos focais e analisados pela técnica da análise de conteúdo.

**Resultados:**

As enfermeiras destacaram a autonomia reprodutiva, com aumento na demanda por laqueaduras; a educação personalizada sobre contracepção; o impacto socioeconômico no acesso do planejamento familiar; e desafios relacionados à inclusão masculina, com estratégias voltadas à corresponsabilidade no planejamento.

**Considerações finais:**

Enfermeiras desempenham papel essencial na promoção dos direitos reprodutivos. Pelo respeito às individualidades e o contexto social, as enfermeiras promovem a responsabilidade compartilhada e fortalecem a autonomia feminina, as quais estão alinhadas aos Objetivos do Desenvolvimento Sustentável que visam a igualdade de gênero e a redução das desigualdades.

## INTRODUÇÃO

A saúde e o bem-estar social são influenciados de maneira significativa pelos direitos sexuais e reprodutivos, os quais refletem nos Objetivos de Desenvolvimento Sustentável (ODS) estabelecidos pela Organização das Nações Unidas (ONU), que visam a saúde de qualidade (ODS 3), educação de qualidade (ODS 4), igualdade de gênero (ODS 5) e redução das desigualdades (ODS 10)^(1)^. A análise desses direitos sob diferentes perspectivas, incluindo legislação, políticas públicas e movimentos sociais, é crucial para entender os avanços e desafios na efetivação do acesso universal à saúde reprodutiva e à liberdade sexual sem coerção ou discriminação^(2)^.

Os ODS constituem uma agenda global estabelecida pela ONU em 2015, com o propósito de erradicar a pobreza, proteger o meio ambiente e promover o bem-estar e oportunidades para todos de forma justa e sustentável^(1,3)^. Ela compreende 17 metas e 169 alvos, os ODS abrangem desafios críticos como pobreza, saúde, educação, igualdade de gênero, e sustentabilidade ambiental^(1)^. Desse modo, servem como um plano para ações conjuntas entre governos, sociedade civil, setor privado e indivíduos visando construir um mundo mais equitativo e próspero^(1,3)^. Profissionais de saúde, especialmente os da enfermagem, têm um papel essencial na concretização dos ODS devido ao seu compromisso com o cuidado e a saúde global^(4)^.

Os cuidados de saúde, com especial destaque para a atenção primária, desempenham um papel decisivo na efetivação dos direitos reprodutivos, sobretudo, quando os serviços de saúde são inclusivos, equitativos e centrados no usuário. A Lei do Planejamento Familiar de 1996, por exemplo, foi um marco na consolidação do planejamento familiar como direito e na garantia de acesso aos métodos contraceptivos e informações sobre saúde reprodutiva no Brasil^(5)^. Essas iniciativas têm contribuído para a melhoria de indicadores de saúde e bem-estar e para o progresso em direção aos ODS, como a redução das taxas de gravidez não planejada e da mortalidade materna e neonatal, além da prevenção de Infecções Sexualmente Transmissíveis (ISTs) e impactos financeiros nas famílias^(6,7)^.

Contudo, apesar dos avanços, a plena implementação de leis e políticas ainda não é uma realidade em todas as regiões do país, resultando em disparidades significativas no acesso e na garantia dos direitos sexuais e reprodutivos^(8)^. As disparidades sociodemográficas influenciam diretamente a assistência ao planejamento familiar e resultam em um acesso desigual aos serviços de saúde, levando a maiores taxas de gravidez não planejada e ISTs^(9)^. Além disso, questões controversas, como a legalização do aborto e os direitos reprodutivos de pessoas LGBTQIA+, enfrentam resistências políticas e sociais, limitando o acesso a serviços essenciais^(10)^.

As políticas públicas direcionadas à saúde sexual e reprodutiva devem ser desenvolvidas com estratégias descentralizadas, operando no âmbito da Atenção Primária em Saúde (APS) para promover assistência integral e equânime^(11)^. O fortalecimento do planejamento familiar é fundamental para transformar esses direitos em uma realidade acessível a todos, refletindo um compromisso com a saúde integral, a dignidade humana e os princípios dos ODS.

Nesse enfoque, a enfermagem ocupa uma posição estratégica na promoção dos direitos reprodutivos, particularmente no contexto do planejamento familiar. Enfermeiras e enfermeiros são, frequentemente, os primeiros profissionais de saúde a interagir com os usuários, exercem um papel determinante na educação e no aconselhamento, além de proverem serviços essenciais de saúde reprodutiva^(12)^. Tal posição privilegiada possibilita que atuem como defensores dos direitos dos usuários, assegurando o acesso a informações e serviços de qualidade, em respeito à autonomia, às escolhas individuais e avançando no cumprimento dos ODS^(13)^.

Os profissionais da enfermagem também desempenham um papel ativo no fornecimento de cuidados diretos em saúde reprodutiva, o que inclui administração de contraceptivos, realização de exames preventivos, acompanhamento durante a gravidez e o pós-parto, e tratamento de ISTs^(14)^. Portanto, a atuação da enfermagem no planejamento familiar e nos direitos reprodutivos contribui para a promoção da saúde, educação e assistência, sendo essencial para o desenvolvimento de políticas públicas e práticas de saúde baseadas em evidências, moldando, assim, o campo da saúde no país.

## OBJETIVO

Compreender como enfermeiras da atenção primária compreendem e integram os direitos reprodutivos no contexto do planejamento familiar.

## MÉTODO

### Aspectos éticos

O presente estudo seguiu as recomendações da Resolução do Conselho Nacional de Saúde (CNS) n° 466/2012 e foi aprovado pelo Comitê de Ética em Pesquisa da instituição proponente. O Consentimento Livre e Esclarecido foi obtido de todas as participantes envolvidas na pesquisa por meio escrito. Assegurando o anonimato das participantes, suas falas foram identificadas pela letra “E” de Enfermeira, seguida de um algarismo numérico, de E1 a E5.

### Referencial teórico-metodológico

Ancorada nos ODS da ONU, que endereçam desafios como saúde, educação e igualdade de gênero, esta pesquisa enfoca no planejamento familiar na atenção primária à saúde. O planejamento familiar na atenção primária é decisivo para o avanço de metas relacionadas à saúde de qualidade (ODS 3), educação de qualidade (ODS 4), igualdade de gênero (ODS 5) e redução das desigualdades (ODS 10), contribuindo diretamente para a sustentabilidade populacional e o bem-estar das comunidades, alinhando-se assim aos esforços globais para o cumprimento dos Objetivos da Agenda 2030. Profissionais de enfermagem são reconhecidos por sua contribuição estratégica na promoção desses objetivos, sobretudo, no contexto da atenção primária à saúde^(4,15)^.

### Tipo de estudo

Realizou-se uma pesquisa-ação em uma Unidade Básica de Saúde de um município da região central do estado do Rio Grande do Sul. A pesquisa-ação possui caráter participativo, impulso democrático e contribui para a mudança social, desenvolve-se a partir de uma necessidade identificada pelo coletivo, provocadas pelo pesquisador, envolvendo esforços, análises e reflexões dos participantes do contexto observado^(16,17)^. O relatório do estudo seguiu diretrizes do *Consolidated Criteria for Reporting Qualitative Research* (COREQ) para assegurar rigor metodológico e transparência^(18)^.

### Cenário do estudo

Pesquisa realizada em uma Unidade Básica de Saúde (UBS) do centro do estado do Rio Grande do Sul, escolhida por ser o local de trabalho da pesquisadora principal e por oferecer um cenário favorável para a investigação, em virtude da alta prevalência de gravidez não planejada e curtos intervalos intrapartos. Para minimizar o risco de viés, a pesquisadora principal seguiu as diretrizes do COREQ (Domínio 1 - Relacionamento com os participantes), mantendo uma postura reflexiva e assegurando que a dinâmica dos grupos focais fosse facilitada por um moderador independente. A pesquisadora obteve autorização da gestão para implementar a consulta de enfermagem em planejamento familiar.

A referida UBS conta com duas Equipes de Atenção Primária (EAP) e duas equipes de Estratégia de Saúde da Família (ESF). A equipe de enfermagem das EAPs é composta por duas Enfermeiras e quatro técnicos de enfermagem para ambas as equipes; e a ESF dispõe de duas enfermeiras e três técnicos de enfermagem. A UBS também conta com uma Enfermeira residente do primeiro ano em enfermagem obstétrica e uma docente de enfermagem de duas universidades do Rio Grande do Sul. Portanto, a equipe é composta por seis enfermeiras, sendo uma delas a pesquisadora.

### Fonte de dados

Todas as cinco enfermeiras da UBS - uma residente, uma docente e enfermeiras das equipes da Atenção Primária e da Estratégia de Saúde da Família - participaram da pesquisa. Foram critérios de inclusão: atuar nas equipes da UBS e dispor de tempo para participar dos três encontros focais. Já os critérios de exclusão seguem: encontrar-se em esquema de supervisão, de férias, de atestado médico e/ou com indisponibilidade para participar dos três encontros.

As participantes foram abordadas pessoalmente pela pesquisadora principal, que apresentou os objetivos e métodos da pesquisa, explicando a importância da participação de cada uma delas para o estudo. Foram realizadas reuniões iniciais para esclarecer dúvidas e obter o consentimento livre e esclarecido das enfermeiras. Tal abordagem direta facilitou a criação de um ambiente de confiança e colaboração entre a pesquisadora e as participantes.

### Coleta e organização dos dados

Os dados foram coletados por meio da técnica de grupo focal (GF), que possibilita discussões aprofundadas relacionadas a uma determinada temática de interesse comum dos participantes^(19)^. Foram realizados três encontros focais entre os meses de setembro e novembro de 2023, com a duração em média de uma hora e meia cada encontro. Os dias e horários dos encontros foram agendados previamente com os participantes e realizados após o turno laboral vespertino.

Os encontros foram realizados em uma sala de reuniões da UBS, com a organização das cadeiras em círculo, de modo a favorecer a discussão e a interação entre os participantes. A sistematização dos encontros focais contou com a participação da pesquisadora principal, na função de coordenação; um moderador, responsável pelos debates junto à coordenação; e de duas bolsistas de iniciação científica, previamente treinadas e encarregadas pela gravação em áudio por um smartphone (*Iphone* 8 *Plus*) e anotações.

O grupo focal foi conduzido com base em um guia disparador do debate, que incluía perguntas a exemplo de: como vocês compreendem e integram os direitos reprodutivos no contexto do planejamento familiar? Quais são os principais desafios que vocês enfrentam ao promover esses direitos? De que maneira as condições socioeconômicas das usuárias influenciam o acesso ao planejamento familiar? Que estratégias vocês utilizam para personalizar as orientações sobre métodos contraceptivos? Como as mudanças legislativas recentes têm impactado o trabalho de vocês em relação aos direitos reprodutivos?

A clareza e a pertinência das questões foram debatidas junto ao Grupo de Pesquisa, composto pela pesquisadora principal da pesquisa (enfermeira), um pesquisador auxiliar (enfermeiro), uma pesquisadora doutora (enfermeira), uma bolsista de iniciação científica (acadêmica de psicologia) e a orientadora da pesquisa (psicóloga).

Durante os encontros, as informações mencionadas foram verificadas e registradas, com uma leitura final realizada ao término de cada encontro, permitindo que as participantes tivessem a oportunidade de complementar e esclarecer alguma informação e sobretudo recebessem os resultados da pesquisa.

### Análise dos dados

Os dados gravados foram transcritos na íntegra e submetidos à Análise de Conteúdo^(20)^, seguindo as etapas de pré-análise, exploração do material e tratamento dos resultados. Na pré-análise, realizou-se a leitura fluente dos dados coletados, em que se buscou uma primeira impressão e representação das mensagens; na etapa da fase exploratória, o material foi codificado em categorias por meio de recortes dos registros; já na terceira e última etapa, a partir do tratamento dos resultados, os dados foram tratados de maneira que pudessem ser significativos, por meio de avaliação crítica e reflexiva.

## RESULTADOS

O estudo resultou na participação de cinco enfermeiras, com idade entre 25 e 48 anos. O tempo de atuação profissional na UBS variou entre 11 meses e 15 anos. Todas possuem pós-graduação lato sensu, do tipo especialização e duas pós-graduação stricto-sensu, mestrado e doutorado, respectivamente. Duas enfermeiras atuam, ainda, em outro serviço na assistência hospitalar.

Dos dados organizados e analisados, emergiram três categorias: Educação em saúde sobre saúde sexual e reprodutiva; Vulnerabilidade social e direitos reprodutivos; e Percepções e contribuições das enfermeiras para os direitos reprodutivos.

### Educação em saúde sobre saúde sexual e reprodutiva

Nesta categoria, emergiram dados sobre como as enfermeiras promovem a educação em saúde, com enfoque na personalização das orientações, diversificação dos métodos contraceptivos e inclusão do planejamento familiar em diferentes contextos de cuidado.

*Durante a consulta de Enfermagem, falamos sobre o que tem disponível, vemos a preferência da paciente e as possibilidades. Tem a questão da elevada incidência das que perdem o prazo de fazer aquele injetável, então precisamos fazer o teste rápido de gravidez, ou um BHCG. Então, considerando demanda espontânea, seria nesse momento que a gente faria uma conversa, uma orientação, sobre essa temática.* (E2)

*No pré-natal, no último trimestre, quando a gente começa a falar sobre o planejamento familiar, a gente conversa sobre os projetos de vida delas, se ter uma outra gestação é um projeto, para logo ou não, e sobre métodos contraceptivos possíveis de ser utilizado, se elas tiverem aleitamento materno exclusivo ou não, no caso de uma gestante com HIV. São nessas questões que a gente foca quando pensa no planejamento familiar.* (E1)

*Eu acho que a minha fala está voltada mais para essa parte* [de contracepção], *de orientar, de perguntar se tem algo da preferência. A consulta de Enfermagem, quando você está fazendo um teste rápido, você está perguntando para a mulher como é que está o seu preventivo, ou se faz uso de algum método. Às vezes vem com alguma queixa voltada mais ao climatério e menopausa. A consulta de Enfermagem é muito ampla.* (E2)

Desafios em comunicação e compreensão sobre métodos contraceptivos foram especialmente notados em contextos de vulnerabilidade psicossocial.

*As questões psicológicas e sociais, porque tem algumas que não vão aderir aquele método, mesmo que explique, explique, explique. Às vezes tu tens que dar uma atenção maior, porque elas* [usuárias] *não têm aquela capacidade de conseguir entender o tanto quanto que a gente acha que passa para elas. Então isso tem muito, a gente acha que elas entenderam e elas chegam na outra consulta e não entenderam nada de como elas usam a pílula, ou o que aquilo vai causar para a vida delas.* (E5)

### Vulnerabilidade social e direitos reprodutivos

As enfermeiras destacaram como a vulnerabilidade social afeta o acesso e a efetividade do planejamento familiar, reconhecendo a inter-relação entre condições socioeconômicas e direitos reprodutivos.

*Eu acho que quando falamos na questão de planejamento familiar,* [...] *pensamos na importância, enquanto profissionais que atuam na saúde da mulher, de falar sobre esses direitos sexuais e reprodutivos, e que existe uma questão importante* [...] *que é a vulnerabilidade, a situação de vulnerabilidade a que a mulher está exposta* [...] *essa vulnerabilidade que é individual, que é social, que é usuário de droga, que sofre violência, enfim, que essas questões também implicam no planejamento familiar.* (E1)

*Acho que, devido à vulnerabilidade, as pessoas acham que é normal perder vários filhos* [...] *e não procuram ajuda antes, e isso também reflete um pouco no planejamento familiar como um todo, porque também não procuram ajuda quando precisam prevenir a gravidez.* [...] *elas não procuram, porque elas têm essa vulnerabilidade social e familiar também.* (E5)

Discussões sobre as dimensões socioeconômicas sublinharam o desafio contínuo na promoção dos direitos reprodutivos.

*Sabemos das questões de vulnerabilidade em relação à pobreza, mesmo, né? Poucos recursos financeiros.* (E3)

*Nossas mulheres e nossas crianças são muito vulneráveis.* [...] *Chegou um e-mail para acompanhar um caso de uma moradora de rua.* (E4)

*A gente teve um caso, ela teve 4 abortos e ela queria, planejava muito e queria muito uma gestação. A gente sabe das questões de vulnerabilidade em relação à pobreza mesmo* [...]*. Mas tem os direitos reprodutivos, ela queria muito ser mãe e ela tinha também esse direito. Por mais que, às vezes, a gente não concorde com elas, pois sabemos que, daqui a pouco, vai expor a uma maior vulnerabilidade, elas têm que ter o direito.* (E5)

### Percepções e contribuições das enfermeiras para os direitos reprodutivos

As contribuições das enfermeiras para a garantia dos direitos reprodutivos foram evidenciadas, abordando tanto as mudanças legislativas quanto a autonomia das mulheres em suas decisões reprodutivas.

*A gente sabe que muitas mulheres* [...] *ainda estão sujeitas às decisões de um homem, então elas estão tendo essa autonomia que agora é respaldada pela alteração da lei.* [...] *Tem mais procura, porque agora se eu tenho 21, se eu tenho dois filhos, eu tenho autonomia. Antes eu dependia do aval do meu parceiro, enfim, ficava truncado.* (E2)

*A gente tem várias situações que é um filho de cada parceiro, que é o parceiro do momento, então ficavam reféns porque não preenchiam os requisitos* [para laqueadura]*, e no meu ver não é justo isso aí, porque o corpo é dela, ela* [a mulher] *que tem que decidir.* (E4)

*Está grávida de um estupro vamos ver se ela vai ter acesso a esse planejamento, essa laqueadura que ela tanto quer, porque a lei como sempre tudo é muito bonito* [...]*. Se fosse antigamente ela ia ter que procurar este homem, para dar ‘ok’, imagina ter que procurar o estuprador dela para conseguir uma permissão.* (E4)

*Eu falo também dos métodos, quando é a questão de gestante, a gente fala depois de um DIU* [Dispositivo Intrauterino]*, de uma laqueadura, se tem vontade. A gente fala um pouco com o marido* [...] *então avalia isso, fala dos métodos.* (E5)

*Está tendo muita procura da laqueadura e já estamos encaminhando também para fazer laqueadura e vasectomia. Para não só sobrecarregar a mulher, porque sempre é a mulher, né? Então, já faz encaminhamento pra saúde da mulher, já faz orientações com os dois, para corresponsabilizar, pois o planejamento familiar não é algo só da mulher.* (E4)

*No momento a gente está fazendo mais encaminhamentos para laqueadura, acho que é em função da legislação que ficou mais facilitadora.* (E2)

## DISCUSSÃO

### Educação em saúde sobre saúde sexual e reprodutiva

Na APS, as enfermeiras desempenham um papel central na educação e comunicação sobre saúde sexual e reprodutiva. As consultas de enfermagem revelam uma abordagem estratégica que enfatiza a individualização do atendimento e o suporte contínuo ao uso de métodos contraceptivos. Nas consultas de rotina e sessões pré-natais, emergem oportunidades para discutir o planejamento familiar e selecionar métodos contraceptivos adequados

A orientação individualizada, ilustrada pelas enfermeiras E2 e E1, destaca a necessidade de uma abordagem personalizada, que envolve a exploração das opções de contracepção, o entendimento das preferências dos usuários e o acompanhamento da adaptação aos métodos escolhidos. A prática reflete uma base de conhecimento, experiência e respeito mútuo, essenciais para a construção de uma relação de confiança e uma comunicação eficaz em saúde sexual^(21)^.

Ao considerar as circunstâncias individuais, como amamentação ou condições específicas como o HIV, a ênfase nos projetos de vida é determinante para assegurar os direitos reprodutivos, reforçando a literatura que enfatiza o papel do enfermeiro como mediador entre a comunidade e os serviços de saúde^(22)^. Independentemente da abordagem metodológica utilizada, atividades educacionais devem fomentar a participação ativa e a troca de informações, fortalecendo os vínculos entre usuários e profissionais de saúde^(23)^. O enfoque colaborativo e a abordagem integral é fundamental para uma assistência integral à saúde da mulher^(24)^, mantendo-se como um componente vital para alcançar os ODS, bem como o conhecimento necessário para tomar decisões informadas sobre suas vidas sexuais e reprodutivas, impactando positivamente na sua autonomia, qualidade de vida e consequentemente na saúde das mulheres.

Fatores socioeconômicos adversos, baixos níveis educacionais e uma percepção de status social diminuída podem resultar em um letramento em saúde reduzido. Tais circunstâncias exigem dos profissionais uma atenção redobrada e estratégias de comunicação reforçadas para assegurar a compreensão e o uso adequado dos métodos contraceptivos^(25,26)^.

A atuação das enfermeiras na educação em saúde sexual e reprodutiva vai além da transmissão de informações, incluindo a compreensão das circunstâncias individuais. Para enfrentar os desafios da comunicação eficaz, é necessário um acompanhamento personalizado, especialmente em contextos de vulnerabilidade^(27)^.

### Vulnerabilidade social e direitos reprodutivos

A vulnerabilidade social inclui pessoas com as mesmas peculiaridades, isto é, suscetíveis a sofrer algum tipo de lesão além das físicas e morais, sabe-se que mulheres em vulnerabilidade social tem seus direitos (re)negados diariamente. Nesse sentido, torna-se necessário a efetividade das políticas públicas e afirmativas para mulheres, acarretando na nova forma de vislumbrar a mulher, cidadã, inserida na família e na sociedade, sendo detentora de direitos, obrigações, prerrogativas e de deveres^(28)^.

A vulnerabilidade social, marcada pela negação de direitos, impacta a capacidade das mulheres de tomar decisões informadas sobre saúde sexual e reprodutiva. A normalização de eventos adversos, como abortamento e mortalidade infantil, bem como o acesso tardio aos serviços de saúde revelam a necessidade de estratégias educacionais adaptadas às condições socioeconômicas das mulheres^(9)^. A vulnerabilidade social aliada aos direitos reprodutivos pode impactar significativamente as práticas de saúde, criando barreiras que dificultam o acesso a cuidados de saúde. Essas barreiras perpetuam um ciclo de desigualdade que não só afeta a saúde das mulheres, mas também impede o progresso em direção aos ODS. Abordar essas vulnerabilidades requer uma abordagem intersetorial e integrada, que envolva a redução das desigualdades sociais e econômicas, promoção da igualdade de gênero e o fortalecimento de parcerias para garantir que os usuários dos serviços de saúde tenham capacidade de fazer escolhas informadas em conformidade com sua realidade.

A dificuldade no acesso a informações e serviços de saúde reflete uma barreira ao exercício dos direitos reprodutivos, especialmente em grupos vulneráveis, em que ações de planejamento familiar precisam ser intensificadas, o que requer um trabalho de fortalecimento educacional através da APS^(29)^. As participantes do estudo problematizam como as temáticas de cunho sociocultural impactam na saúde e mencionam que, entre a população atendida, há situações de violência, machismo, uso de drogas, assim, elas reconhecem como essas situações devem ser consideradas relevantes. Isso evidencia a necessidade de fortalecer as políticas sociais na área da saúde e que os desafios estão em vários níveis onde as ações do poder público devem responder à promoção integral dos direitos sexuais e reprodutivos da população^(30)^. O direito à maternidade, mesmo em contextos de vulnerabilidade, é uma questão ética, sendo dever do Estado garantir proteção social e condições adequadas de cuidado em saúde^(31)^.

### Percepções e contribuições das enfermeiras para os direitos reprodutivos

O estudo evidencia a percepção das enfermeiras de que o planejamento familiar é essencial para assegurar os direitos reprodutivos das mulheres, destacando a autonomia feminina nas decisões relativas à saúde reprodutiva como um direito fundamental. Alterações legislativas recentes, em consonância com tratados internacionais, ampliaram o acesso das mulheres a esses direitos, aumentando a demanda por procedimentos reprodutivos e refletindo a progressiva emancipação feminina^(32)^. Tais percepções corroboram com um estudo que identificou o aumento do número de laqueaduras realizadas entre 2019 e 2022, além de um crescimento adicional em 2023, atestando que a aprovação da nova lei da laqueadura atribuí empoderamento às mulheres em relação aos seus direitos reprodutivos^(33)^.

As mudanças na legislação ressaltam a transição histórica rumo à igualdade de gênero, eliminando requisitos obsoletos que restringiam a autonomia das mulheres, como a autorização conjugal para procedimentos de laqueadura^(34)^. O tema envolve ética, política, religião, costumes, questões sociais e demográficas, pautas de discussões em cenário mundial. As alterações feitas na Lei 14.443 expressam um marco nos movimentos sociais em busca de maior autonomia das mulheres e a busca pela igualdade de gêneros. Em especial, a revogação da exigência do consentimento expresso pelo cônjuge que representa um grande avanço na conquista social das mulheres^(35)^.

No entanto, persistem desafios na educação em saúde e no acesso equitativo aos serviços de planejamento familiar, com as enfermeiras enfatizando a necessidade de envolver os homens nas consultas para promover a corresponsabilidade. A literatura sugere que os índices dos métodos contraceptivos com maior representatividade de escolha social é laqueadura de trompas e que a preferência pode estar relacionada com a falta de educação em saúde, problemas com a cobertura e na captação do público masculino nos serviços de planejamento reprodutivo^(36,37)^. Ações conjuntas são fundamentais para assegurar a saúde e o bem-estar reprodutivos do casal, com o Sistema Único de Saúde (SUS) oferecendo métodos contraceptivos específicos para homens, como vasectomia e preservativos masculinos, promovendo a equidade de gênero no planejamento reprodutivo^(38-40)^.

É imperativo adotar uma abordagem ampliada que considere a saúde sexual e reprodutiva ao longo de todo o ciclo de vida, envolvendo ativamente os homens para superar as desigualdades de gênero e promover a corresponsabilidade^(41)^. Essas percepções corroboram com outro estudo, em que as Enfermeiras entrevistadas também percebem que as mulheres são vítimas das desigualdades de gênero, da responsabilização exclusiva pela proteção contra IST e das medidas contraceptivas, com consequente falta de acompanhamento do parceiro e culpabilização das mulheres pelas gestações indesejadas^(42)^. A responsabilidade parental conjunta tem efeitos benéficos sobre o desenvolvimento das crianças e o fortalecimento dos laços familiares, com os profissionais de saúde incentivando a participação masculina desde as consultas pré-natais até os cuidados pós-parto^(39)^.

Ainda assim, as percepções das enfermeiras demonstram uma matriz cisheteronormativa, com lacunas no atendimento às necessidades de pessoas LGBTQIA+, que enfrentam barreiras adicionais no acesso a serviços de planejamento familiar. É necessário ampliar as abordagens para incluir uma compreensão diversa das identidades de gênero e orientações sexuais, garantindo que os direitos reprodutivos sejam acessíveis a todos. Mesmo com os problemas de contexto social, estrutural, humano, financeiro e de gestão, ainda assim a atuação do enfermeiro se destaca como o profissional essencial para promoção da saúde e atendimento integral da população^(43)^. Ainda assim, todos esses achados evidenciam ser indiscutível a necessidade de ações do Estado para a garantia dos direitos reprodutivos, ao proteger a saúde e a autonomia das pessoas, promover a igualdade de gênero e eliminar barreiras legais e sociais que possam limitar o acesso a esses direitos.

### Limitações do estudo

A pesquisa foi realizada em apenas uma Unidade de Saúde, limitando o número de participantes e a extensão dos resultados para outros contextos. Fatores culturais e contextuais específicos devem ser considerados na transposição dos resultados para diferentes realidades.

### Contribuições para a área da enfermagem, saúde materno-infantil e políticas públicas

A pesquisa sugere melhorias nas políticas públicas, com destaque para a inclusão dos homens no planejamento familiar e a corresponsabilidade na contracepção, alinhadas aos ODS. Os profissionais da enfermagem são fundamentais para implementar essas mudanças, influenciando a formulação de políticas eficientes. As estratégias educacionais e políticas públicas que promovam os direitos reprodutivos reforçam o papel estratégico da enfermagem na promoção da saúde e bem-estar, em conformidade com os princípios dos ODS.

## CONSIDERAÇÕES FINAIS

Essa pesquisa proporcionou uma análise qualitativa acerca da compreensão e integração dos direitos reprodutivos por enfermeiras atuantes na atenção primária, no âmbito do planejamento familiar. As profissionais evidenciaram a necessidade de individualizar o cuidado à saúde feminina, enfatizando a importância de abordar tais temas de maneira personalizada, respeitando as preferências e necessidades dos usuários. Salientaram-se os desafios comunicacionais e de compreensão, onde o aprimoramento do letramento em saúde surge como estratégia para assegurar a clareza e acessibilidade das informações.

Reconheceu-se a influência da vulnerabilidade social nos direitos reprodutivos, destacando-se que as condições socioeconômicas são determinantes para a promoção da equidade no acesso aos cuidados de saúde reprodutiva. As enfermeiras observaram que as recentes reformas legislativas têm fortalecido a autonomia reprodutiva feminina, refletida no aumento da demanda por procedimentos como a laqueadura. Entretanto, identificam-se obstáculos na operacionalização dessas políticas, sublinhando a necessidade de atender às expectativas femininas. A predileção por métodos contraceptivos específicos aponta para lacunas na educação em saúde e desafios na inclusão masculina em serviços de planejamento reprodutivo. Ressalta-se a relevância de integrar os homens nas consultas de planejamento familiar, visando uma distribuição equitativa das responsabilidades contraceptivas.

Por fim a presente investigação destaca a relevante contribuição das enfermeiras da atenção primária na promoção dos ODS, com ênfase nos objetivos que abordam a saúde de qualidade (ODS 3), educação de qualidade (ODS 4), igualdade de gênero (ODS 5) e a redução das desigualdades (ODS 10). Por meio da promoção da autonomia das mulheres, respeito às individualidades e incentivo à responsabilidade compartilhada na contracepção, as enfermeiras desempenham um papel fundamental na implementação de práticas alinhadas com as metas globais para o desenvolvimento sustentável. Sublinha-se a importância de políticas públicas robustas e de iniciativas educacionais focadas no fortalecimento da capacitação em direitos reprodutivos, para profissionais de saúde e membros da comunidade, reforçando assim o papel estratégico da enfermagem na obtenção desses objetivos globais.

## Data Availability

Os dados de pesquisa estão disponíveis estão no corpo do artigo.
